# Pennsylvania Hospital

**Published:** 1838-08-01

**Authors:** 


### CLINICAL REPORTS.

### PENNSYLVANIA HOSPITAL.

*Case of gunshot wound of the hand, healed by the first intention.*

ALEXANDER ——, æt. seven years, was brought to the Hospital, July 5th, with a lacerated wound of the right hand, caused by the discharge of a gun, loaded with powder and paper, while his hand was over the muzzle. The space, between the first and second metarcarpal bones, was completely laid open, and the skin torn up for a distance of about two inches, on the upper part of the hand. The palmar arch was, of course, divided, but there was no hæmorrhage, owing to the cauterization of the blazing powder. A small superficial vessel was secured, the wound drawn together with adhesive straps, dressed with dry lint, and placed on a splint, and an opiate given.

*July 6th.*—Slight fever—laxative and low diet. *Cold applications were kept constantly on the hand, by means of a syphon, for twelve days*, during which time the dressings were not disturbed, the child complaining of no pain.

*July 17th.*—The dressings were removed for the first time since the accident, and the wound was found to have united by the first intention, excepting at the surface, where the skin had been lacerated. Nitrate of silver to the edges of the wound, which were brought together with adhesive straps, a dressing of simple cerate, and the hand was laid on a splint.

*August 1st.*—The wound has been dressed daily, the edges of the sore touched with nitrate of silver, and it is now completely cicatrized.

This case illustrates the happy effects of the application of cold to lacerated wounds, now practised to a considerable extent in Paris, in place of the ordinary warm poultices which have been, heretofore, usually recommended. A gunshot wound, of sufficient severity to render the loss of the hand not improbable, in this instance, healed in a very short time, without pain; while, had heat been applied to it, it must have sloughed, with danger of secondary hæmorrhage, and other bad consequences.

*List of Accidents, admitted into the Pennsylvania Hospital, from July 11th to July 25th, 1838.*

One case of dislocation of the humerus into the axilla, caused by a fall upon the shoulder; reduced at once, by extension with a towel and wet roller, and the heel in the axilla.—One case of compound, comminuted fracture of the skull, under the right temporal muscle, close to the junction of the temporal and parietal bones, with depression—the dura mater very slightly wounded; trephined by Dr. NORRIS: ten days after the operation, symptoms of inflammation of the brain, and effusion supervened, the arm, opposite to the side of the head wounded, being paralyzed; the patient has been slightly mercurialized, with great improvement of the symptoms, and gradual subsidence of the paralysis.—One case of incised wound of the arm; hæmorrhage, from some small vessels, was arrested by pressure, and the edges of the wound drawn together by adhesive straps; doing well.—One case of burn, involving the right hand, part of the fore arm, neck, and breast; dressed with poultice; sloughs since separated, and the ulcers are now dressed with Goulard's cerate, and the hand and arm placed on a splint.—One case of oblique fracture of the head of the tibia, from the fall of a bank of earth upon the leg, on the rail-road, near Norristown: the man was brought to the hospital, twenty hours after the accident, with intense inflammation of the knee-joint; V. S. ad ℥xlviij., ad deliquium, and ℥xx. of blood were afterwards taken from the knee, by leeches; low diet and a purge. The patient, a stout, healthy young man, bore the depletion well; the inflammation has very much subsided, and the leg is now treated after the usual mode, by a fracture-box.—One case of fracture of the coracoid process and neck of the scapula, caused by a fall from a waggon upon the shoulder; treated with the clavicle apparatus.—One case of oblique fracture of the middle of the thigh, in a child, eight years of age; treated with Physick's modification of Desault's splints. Excoriation along the course of the counter-extending band, and a tendency to slough in the heel, having appeared, two long splints have been applied to the thigh, nine days after the accident, and the limb is retained in Desault's apparatus, extension being abandoned.—One case of contused ankle, cured by rest and cold applications: the man was threatened with mania a potu, the incipient symptoms of which were relieved by the free use of opiates and tincture of valerian.—One case of poisoning, from the introduction of half an ounce of arsenic into the stomach; immediately after the accident, an emetic, of ipecacuanha and sulphate of copper, had been given to the man, previous to his entrance into the hospital; after his admission, an ounce of the hydrated peroxide of iron, in two doses, at the interval of an hour, was administered. The habits of the patient were of the last degree of intemperance, amounting, on his own admission, to the daily use of a quart of ardent spirits. Symptoms of intense gastritis supervened; thirst and vomiting, every thing being rejected by the stomach, except a little ice. Pulse one hundred and twenty per minute, and very feeble. Brandy and laudanum were freely exhibited by the rectum; but the patient sank, eighteen hours after the arsenic had been swallowed. Examination after death revealed immense inflammation of the mucous membrane of the stomach, extending down into the duodenum, amounting, in places, to the effusion of blood beneath the membrane.—One case of fracture of the upper third of the humerus, accompanied by violent inflammation. Treated at first with cold applications, and an attempt to retain the fragments in apposition by the clavicle apparatus: very severe mania a potu supervened, during which the arm was bandaged tightly in a pillow; the mania a potu has been cured by the free use of brandy and a blister to the back of the head; there is a disposition to slough, about the fracture, which is treated, for the present, by cold applications and three long splints.—One case of oblique fracture of the upper third of the thigh, from the caving in of a bank of earth upon the limb; treated with Physick's and Desault's apparatus; severe mania a potu, with great prostration, has come on, and the patient is at present under a treatment of free stimulation, with a blister to the back of the head.—One case of incised wound of the wrist and palm of the hand, from a cut with a scythe; no large vessel injured, and two small ones were taken up; the wound was drawn together by adhesive straps. The dressings were removed at the end of three days, and the wound found to be nearly united by the first intention.—One case of fracture of the tibia and fibula, just above the ankle joint; treated with the fracture-box, and lead water: mania a potu has since supervened, and the man is at present under a treatment, similar to that employed in the two cases above mentioned, combined with opiates.—One case of lacerated wound of the arm, which had been caught on an iron spike; no hæmorrhage of consequence; the wound was brought together by three stitches, and cold applied to it, by means of a syphon: the dressings have not yet been disturbed.—One case of contusion of the calf of the leg; relieved by rest and cold applications.

The contused wound of the thigh, made by the red hot iron, mentioned in the last number, is nearly well. A counter opening formed in the lower part of the thigh; a free discharge was kept up by pressure, and the wound has granulated, two small superficial ulcers only remaining. The other cases are still under treatment.

